# Is There a Connection Between Mental Health Issues and Poverty? AService Evaluation of East Norfolk/Suffolk Youth Service Patients

**DOI:** 10.1192/bjo.2025.10477

**Published:** 2025-06-20

**Authors:** Dawn Collins, Jo Lowe

**Affiliations:** 1 NSFT, Great Yarmouth, United Kingdom; 2 NSFT, Norwich, United Kingdom

## Abstract

**Aims:** It is well established that living, or growing up, in poverty has a negative impact on both physical and mental health. The area our service covers includes Great Yarmouth and Lowestoft, two of the most economically impoverished areas of the UK. The vast majority of our patient group will have grown up in relative poverty. While there are associations between poverty and impaired physical health and increased risk of some mental health conditions, the actual causal link is unclear.

This evaluation tried to consider the impact of poverty on future mental health, by evaluating current patient case load (this stood at 122 in Feb 2024). We considered all patients, their demographics (age, gender, diagnosis) and the factors listed above. This patient group is young people (18–25 years old), living in this area, under Mental Health Services, with or without a formal mental health diagnosis.

**Methods:** An analysis of current case load, recording demographics and noting diagnoses and factors associated with poverty, specifically:

Parental drug or alcohol abuse.

Parental mental health problems (if these are not well managed).

Early/premature death of a parent.

Exposure to domestic violence.

Physical abuse.

Going into the Care System.

Early drug or alcohol use.

Early separation or loss of a parent.

NB – Many of these factors will affect those who do not grow up in poverty (e.g. domestic violence and physical abuse) but they are noted to have a class and poverty association. Many, if not most, of our patients will have grown up in poverty but their mental illness does not have a specific association with poverty (e.g. OCD, Bipolar disorder).

**Results:** Our findings show that a significant percentage of our patient group have mental health issues directly related to poverty. Total number of patients =122. Number who have a specific factor associated with poverty =56. This equates to 46% of our current caseload. Gender: 35 female (62.5%), male 21 (37.5%).

**Conclusion:** “The poor bear the greatest burden of mental illness” (Office of National Statistics).

It is worth noting that the vast majority of our patient case load grew up in poverty, due to the demographics of the area we work in (a quick analysis suggests about 97% are from working class, impoverished backgrounds). We abandoned recording “parental unemployment” in this analysis, because for all but a few, this was the case. Unemployment is an entrenched issue in this area, with the demise of the shipping and offshore industries, currently standing at 5.4% in Yarmouth and 3.5% in Lowestoft (3) (National average 3.8%). For those that are employed, poverty is a significant issue with many in low paid jobs. I have also not included here factors associated with poverty, such as poor diet, smoking, malnutrition, poor dentition, and obesity, but we know these are the case for many patients seen here.

Recommendations: Given that this is the case, what can we recommend, in term of service planning and delivery? We have multiple issues here that affect our service delivery to this vulnerable patient group: geography (we cover a large geographical area, the need of this population, limited public transport – patient often have to travel some distance to be seen), staff recruitment (it would seem this area holds little appeal for new staff, especially Medics and Psychologists and recruitment uptake is low) and funding (do we need extra funding per head population, as this is such a deprived area?).

